# Loss of insulin-expressing extra-islet cells in type 1 diabetes is accompanied with increased number of glucagon-expressing extra-islet cells

**DOI:** 10.1007/s00428-024-03842-4

**Published:** 2024-06-26

**Authors:** Louise Granlund, Marcus Lundberg

**Affiliations:** Department of Immunology, Genetics and Pathology, Uppsala University, Uppsala, Sweden

**Keywords:** T1D, Extra-islet cells, Glucagon, Insulin

## Abstract

The presence of remaining insulin-positive cells in type 1 diabetes (T1D) is well-known. These cells are part of islets or appear as extra-islet insulin-positive cells scattered in the exocrine parenchyma. The latter are poorly described, and the presence of scattered endocrine cells expressing other islet hormones than insulin has not been explored. This study aimed to compare the extra-islet insulin- or glucagon-positive cells concerning their frequency, transcription-factor expression, and mitotic activity in subjects with and without T1D. Multispectral imaging was used to examine extra-islet cells by staining for insulin, glucagon, ARX, PDX1, and Ki67. This was done in well-preserved pancreatic tissue obtained from heart-beating organ donors with or without T1D. In three T1D donors, lobes with insulin-containing islets (ICI) were found. Within these, a higher frequency of extra-islet insulin-positive cells was observed compared to lobes with insulin-deficient islets (IDI). Increased frequency of glucagon-positive extra-islet cells was observed in donors with T1D (median 53 cells/mm^2^) when compared with non-diabetic donors (11 cells/mm^2^, *p* = 0.004). Proliferating endocrine cells were present in donors with, and without T1D, as demonstrated by Ki67-positive staining (0–3% of the cells expressing insulin or glucagon). The reduced frequency of extra-islet insulin-positive cells in lobes with IDI in donors with T1D suggests that the pathological mechanism causing beta cell demise in T1D affects entire lobes. The presence of an increased frequency of glucagon-positive extra-islet cells supports the notion of a preserved capacity to regenerate the endocrine pancreas in donors with T1D.

**Supplementary Information:**

The online version contains supplementary material available at 10.1007/s00428-024-03842-4.

## Introduction

The symptoms of type 1 diabetes (T1D) emanate from beta-cell loss, but the pathophysiology underlining this beta-cell failure is poorly understood. Remaining insulin-containing islets (ICI) and scattered insulin-positive cells in the exocrine parenchyma can be found in patients with T1D [1–3]. However, the ICI are few, and the frequency of the scattered insulin cells is reduced in T1D compared with subjects without diabetes (controls) [4, 5]. Studies on the replication frequency of islet beta-cells, assessed by staining for Ki67, consistently point towards a low proliferating capacity of these cells, with no difference observed between control and T1D subjects [1, 4–6]. The remaining beta cells in T1D seem to be functional, as most patients with longstanding T1D continue to secrete low levels of insulin which increases in response to a meal [3, 7]. The maintained presence of beta cells in the T1D pancreas implies that these beta cells are either escaping destruction or are continuously regenerated, either through replication and/or neogenesis. The proliferative capacity of beta-cells has been proposed to be underestimated [8], with evidence indicating that existing beta-cells can serve as a reservoir for generating new ones, advocating for proliferation [9]. On the other hand, human duct tissue has been demonstrated to be able to differentiate into islet cells in vitro [10]. However, the specific mechanisms that predominate in humans remain a topic of ongoing debate.

Although extensively studied within islets, insulin-positive cells scattered in the exocrine parenchyma are much less explored. Furthermore, the occurrence of glucagon-expressing extra-islet endocrine cells, their phenotype, and possible proliferation have not been studied. We hypothesized that there would be a difference in phenotype and density of extra-islet endocrine cells between T1D and non-diabetic donors. The aim of this study was to compare the extra-islet insulin- or glucagon-positive cells with regard to their density, transcription-factor expression, and mitotic activity in organ donors with or without T1D.

## Material and methods

### Human pancreatic specimens

Pancreases from heart-beating organ donors treated as intended for organ transplantation were procured through the Nordic Network for Clinical Islet Transplantation (https://nordicislets.medscinet.com/en.aspx).

Consent for organ donation (for clinical transplantation and for use in research) was obtained via online database (https://www.socialstyrelsen.se/en/apply-and-register/join-the-swedish-nationaldonor-register/) or verbally from the deceased’s next of kin by the attending physician and documented in the medical records of the deceased in accordance with Swedish law and as approved by the Swedish Ethical Review Authority (Dnr 2023–01845-01). All tissue included in the study was procured, stored, and analyzed as approved by the Regional Ethics Committee in Uppsala (Dnr 2015/444). The pancreases were dissected, and samples were immediately put in 4% paraformaldehyde. At the time of the study, the biobank contained samples from more than 2000 non-diabetic donors and 20 donors with long-standing T1D. From previous examinations of samples from the donors with long-standing T1D, three samples from three different donors were chosen based on the presence of ICI. Sections from the tail region or distal part of the body were available from ten other donors with long-standing T1D. However, one donor had to be excluded due to insufficient tissue quality. Control donors were matched for sex, age, and BMI to the T1D donors. The characteristics of all donors included in the study can be found in Table 1. The medical records of the donors were not made available to protect the integrity of the deceased person. The samples included in the study were collected between year 2009 and 2020.

**Table 1:** Donor characteristics. *Donor no*., donor number, *Age*, years: age of donor in years at the time of death; *BMI*, body mass index, HbA1c in %. *T1D*, subjects with type 1 diabetes. *CTRL*, controls; subjects without diabetes. *NA*, not available

Donor no	Age, years	BMI, kg/m^2^	Sex	HbA1c, %	Pancreas region
T1D-1	16	21.9	M	NA	Body
T1D-2	36	20.9	F	7.4	Body
T1D-3	59	23.9	F	8.2	Tail
T1D-4	68	30.2	M	8.0	Tail
T1D-5	47	27.6	F	7.4	Tail
T1D-6	50	25.4	F	10.9	Tail
T1D-7	25	22.8	M	8.9	Tail
T1D-8	24	27.5	M	8.3	Tail
T1D-9	25	26.7	F	7.1	Tail
T1D-10	65	24.2	M	NA	Tail
T1D-11	70	26.1	M	7.0	Tail
T1D-12	70	18.7	F	5.5	Body
CTRL-1	19	24	M	5.0	Tail
CTRL-2	36	18.9	F	5.1	Body
CTRL-3	57	23.9	F	5.3	Tail
CTRL-4	69	28.7	M	5.7	Tail
CTRL-5	43	27.3	F	NA	Tail
CTRL-6	54	27.3	F	NA	Tail
CTRL-7	27	26.0	M	5.7	Tail
CTRL-8	36	29.3	M	NA	Tail
CTRL-9	22	21.3	F	4.8	Tail
CTRL-10	66	23.4	M	NA	Tail
CTRL-11	71	28.6	M	5.4	Tail
CTRL-12	70	22	F	5.1	Body

### Multiplex staining

Optimization of the antibodies, sectioning, multiplex staining, and scanning were performed as described in Granlund et. al. [11]. Briefly, formalin-fixed and paraffin-embedded (FFPE) samples were sectioned and stained for PDX1, insulin, glucagon, ARX, and Ki67 (in that order) using Akoyas multiplex system and the Opal 6-Plex Detection Kit (cat. nr. NEL821001KT, Akoya Biosciences, Marlborough, Massachusetts) with the Autostainer BOND RX System from Leica Biosystems (cat. nr. 21.2821, Wetzlar, Germany). All reagents were prepared and diluted in the BOND Titration Container Inserts placed in the BOND Titration Containers (cat. nr. OPT9049, Leica Biosystems); the concentrations of all antibodies (primary and secondary) and the Opals used are presented in Supplementary Table 1. The autostainer was run overnight after which the slides were mounted manually using ProLong Diamond Antifade mounting medium (cat. nr. P36961, Thermo Fisher Scientific, Waltham, Massachusetts). The protocol for the autostainer can be found in the supplementary material.

Validation of the sensitivity in the multiplex stainings has been evaluated previously [11]. This was done by performing immunofluorescent stainings (IF) using the same primary antibodies as for the multiplex stainings.

### Scanning and analysis

The slides were scanned using the Vectra Polaris multispectral imaging and whole slide scanning system (recently rebranded to *PhenoImager, Akoya Bioscences*). The analysis was done using the software QuPath (0.3.2), and 200 extra islet cells expressing insulin, glucagon and/or PDX1, and ARX were manually marked and tagged using the polygon tool, and annotated according to the expression profile of that specific cell. The area of the analyzed part of the tissue section was measured using the same tool. In subjects with T1D, all insulin-expressing extra-islet cells within the entire tissue section were counted and annotated. In three T1D donors, areas with ICI were present. To investigate this further, cells were annotated in these donors in areas with and without ICI separately. Cells positive for ARX only, or ARX and PDX1, without any hormone, were not further analyzed, leaving on average 183 annotated hormone-positive cells per donor for analysis (Table 2). Single extra islet cells, as well as small groups of up to four cells, interspersed in the exocrine parenchyma were annotated. Their localization was also noted and defined as (1) peri-islet: being close to an islet (distance 0–3 cells) but outside of the islet border, (2) peri-ductal: being close to a duct (distance 0–3 cells) but not within the ductal epithelium, and (3) intra-acinar: surrounded by acinar tissue and not being defined as peri-islet or peri-ductal (Supplementary Fig. 1). As no staining of ducts was performed, the extra-islet cells defined as being peri-ductal were restricted to extra-islet cells found adjacent to larger ducts that were morphologically distinguishable within the exocrine parenchyma. When several single cells and/or groups of 2–4 cells located close together in a cluster (but not in direct contact with each other) were found, this was also noted.

**Table 2:** The proportion of extra-islet cell-phenotypes in each donor. Extra-islet cells expressing insulin or glucagon were annotated in each donor. The proportion (%) of all phenotypes based on combinations of insulin, glucagon, PDX1, and ARX in each donor is illustrated. In the insulin-containing lobes of two T1D donors (donor nos. T1D-9 and T1D-12), only 81 (donor no. T1D-9) and 62 (donor no. T1D-12) extra-islet cells were found in total. T1D-X INS −  = area with islets devoid of insulin. T1D-X INS +  = area with insulin containing islets. *T1D*, subjects with type 1 diabetes. *CTRL*, controls; subjects without diabetes. *INS*, insulin; *GCG*, glucagon

	Single-hormone positive cells								Double-hormone positive cells				
Donor no	**GCG + **INS-PDX1-ARX-	**INS + **GCG-PDX1-ARX-	**GCG + **INS-**ARX + **PDX1-	**INS + **GCG-**PDX1 + **ARX-	**GCG + **INS-**PDX1 + **ARX-	**INS + **GCG-PDX1-**ARX + **	**GCG + **INS-**PDX1 + ARX + **	**INS + **GCG-**PDX1 + ARX + **	**INS + GCG + **PDX1-ARX-	**INS + GCG + PDX1 + **ARX-	**INS + GCG + **PDX1-**ARX + **	**INS + GCG + PDX1 + ARX + **	**Total no of cells**
T1D-1	71	0	29	0	0	0	0	0	0	0	0	0	194
T1D-2	45	13	29	11	3	0	0	0	0	0	0	0	191
T1D-3	90	1	10	0	0	0	0	0	0	0	0	0	194
T1D-4 INS +	75	8	12	2	1	0	0	0	3	0	0	0	194
T1D-4 INS-	91	1	9	0	0	0	0	0	0	0	0	0	196
T1D-5	74	2	24	0	0	0	0	0	0	0	0	0	193
T1D-6	84	0	16	0	0	0	0	0	0	0	0	0	190
T1D-7	94	0	5	0	1	0	0	0	0	0	0	0	199
T1D-8	59	0	35	0	6	0	0	0	0	0	0	0	194
T1D-9 INS +	19	30	23	26	0	1	0	0	0	0	0	0	77
T1D-9 INS-	56	9	22	12	2	0	0	0	0	0	0	0	180
T1D-10	92	0	7	0	1	0	1	0	0	0	0	0	198
T1D-11	56	4	32	4	4	0	0	0	0	0	0	0	197
T1D-12 INS +	73	6	21	0	0	0	0	0	0	0	0	0	62
T1D-12 INS-	68	0	31	1	2	0	0	0	0	0	0	0	200
CTRL-1	47	21	24	5	1	1	0	0	2	0	0	0	188
CTRL-2	22	37	19	19	2	0	1	0	1	0	0	0	190
CTRL-3	42	42	4	11	0	0	0	0	2	0	0	0	197
CTRL-4	19	55	4	18	1	0	0	1	2	1	0	0	195
CTRL-5	13	63	0	23	0	0	0	1	0	0	0	0	197
CTRL-6	16	54	4	23	1	1	0	1	1	0	0	0	189
CTRL-7	37	45	13	5	0	0	0	0	0	0	0	0	191
CTRL-8	18	41	16	25	0	0	0	0	0	0	0	0	197
CTRL-9	16	33	19	26	0	2	1	1	1	0	1	0	156
CTRL-10	52	18	6	15	1	1	0	1	6	0	1	0	192
CTRL-11	21	50	3	27	0	0	0	0	1	0	0	0	200
CTRL-12	18	37	23	22	0	0	0	0	0	0	0	0	188

### Statistics

Graphpad version 9.5.0 (730) was used to visualize the data, and the Mann–Whitney test was used for comparisons between the groups of subjects with or without T1D. A *p* value of < 0.05 was considered statistically significant.

## Results

### There were fewer insulin- but more glucagon-positive cells among the extra-islet endocrine cells in T1D

**Fig. 1:**
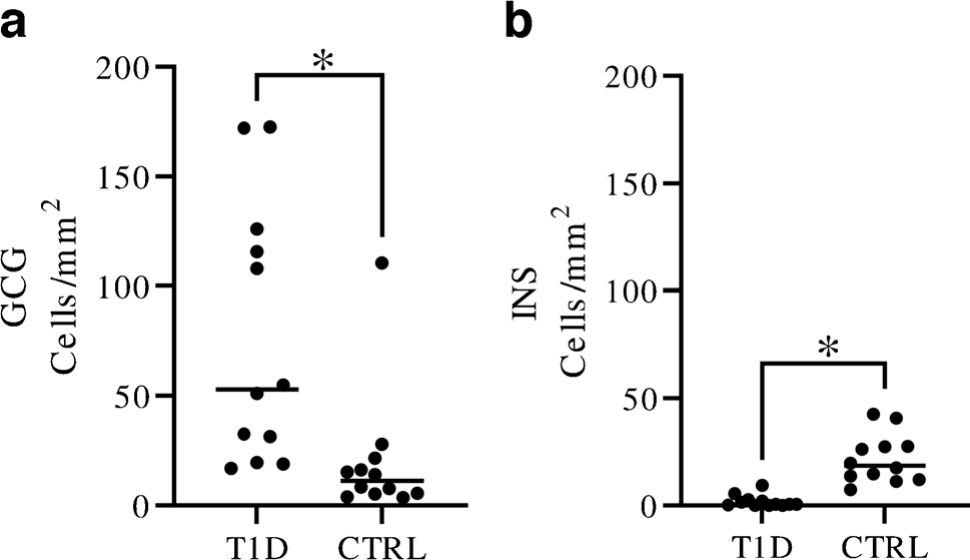
Frequency of extra-islet glucagon- and insulin-positive cells in donors with and without T1D. The frequency of glucagon-positive cells (GCG) per mm^2^ (median T1D = 53.0, median CTRL = 11.2) (**a**). The frequency of insulin-positive cells (INS) per mm^2^ (median T1D = 0.585, median CTRL = 18.6). All insulin-positive cells in the entire tissue section of the T1D donors were annotated (**b**). Each dot represents an individual donor, horizontal line = median. Significance was calculated with a nonparametric Mann–Whitney test. *denotes a significance of < 0.05. T1D, subjects with type 1 diabetes. CTRL, controls; subjects without diabetes

**Fig. 2:**
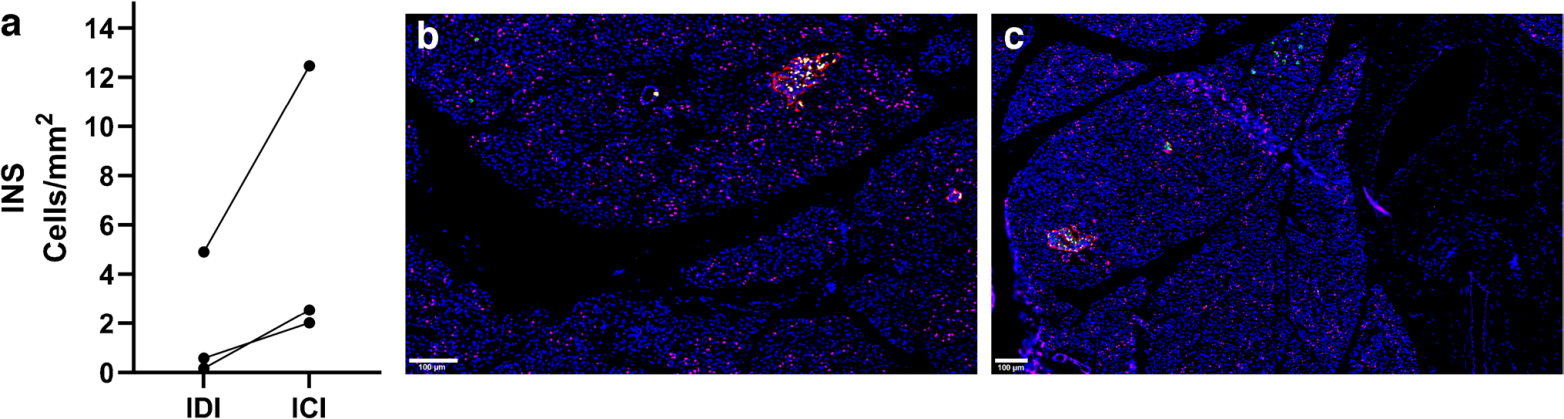
Comparison of extra-islet INS + cells in ICI and IDI lobes of three T1D donors. (**a**) The number of insulin-positive extra-islet cells in ICI and IDI lobes was counted. Interconnected dots illustrate paired samples from the same donor. (**b**–**c**) Representative images of regions with ICI and IDI in a T1D donor. (**b**) Area with IDI—two islets consisting mainly of glucagon-positive cells with no insulin-positive cells are seen. Extra-islet insulin-positive cells are also observed. (**c**) Area with ICI—two islets consisting of both glucagon- and insulin-positive cells are seen. Many extra-islet insulin-positive cells are observed as well. T1D, subjects with type 1 diabetes; ICI, insulin-containing islets; IDI, insulin-deficient islets. Scale bar 100 µm

No difference could be determined in the total frequency of extra-islet cells positive either for insulin or glucagon in donors with or without T1D (64 cells/mm^2^ in the T1D group, range 17–173 cells/mm^2^, and 31 cells/mm^2^ in the control group, range 13–151 cells/mm^2^, *p* = 0.11). Increased frequency of glucagon-positive extra-islet cells was observed in donors with T1D (median 53 cells/mm^2^) when compared with non-diabetic donors (11 cells/mm^2^, *p* = 0.004, Fig. 1a). Conversely, a decreased frequency of insulin-positive extra-islet cells was observed in donors with T1D (median 0.58 cells/mm^2^) when compared with non-diabetic donors (19 cells/mm^2^ *p* =  < 0.0001, Fig. 1b). Extra-islet insulin-positive cells were found in all but one T1D donor (donor no. T1D-10). In three donors with T1D (donor no. T1D-4, T1D-9, and T1D-12), areas with ICI were present. Many of these islets had a normal phenotype, although islets with intra-islet hemorrhages were found in two of the donors (donor nos. T1D-4 and T1D-9). ICI, with and without intra-islet hemorrhages, were located in lobes adjacent to lobes with islets completely devoid of insulin, i.e., IDI. In the lobes with ICI, extra-islet insulin-positive cells were 2.5 to 15 times more frequent than in the lobes with IDI (range between 2 and 13 cells/mm^2^ compared with 0.16–5 cells/mm^2^, Fig. 2). However, in two of the three T1D donors with ICI, only 81 (donor no. T1D-9) and 62 (donor no. T1D-12) extra-islet cells were found in total. The frequency of insulin-positive extra islet cells in the ICI regions was in most cases less frequent than in the control donors (median CTRLs 18.6 cells/mm^2^, range CTRLs 7.4 – 42.5 cells/mm^2^, Table 2). However, there were also a few T1D donors with a higher frequency of insulin-positive extra-islet cells despite no presence of ICIs (Table 2), but less frequent than in any of the control donors.

### No difference in extra-islet endocrine Ki67-expression between T1D and control donors could be determined

**Fig. 3:**
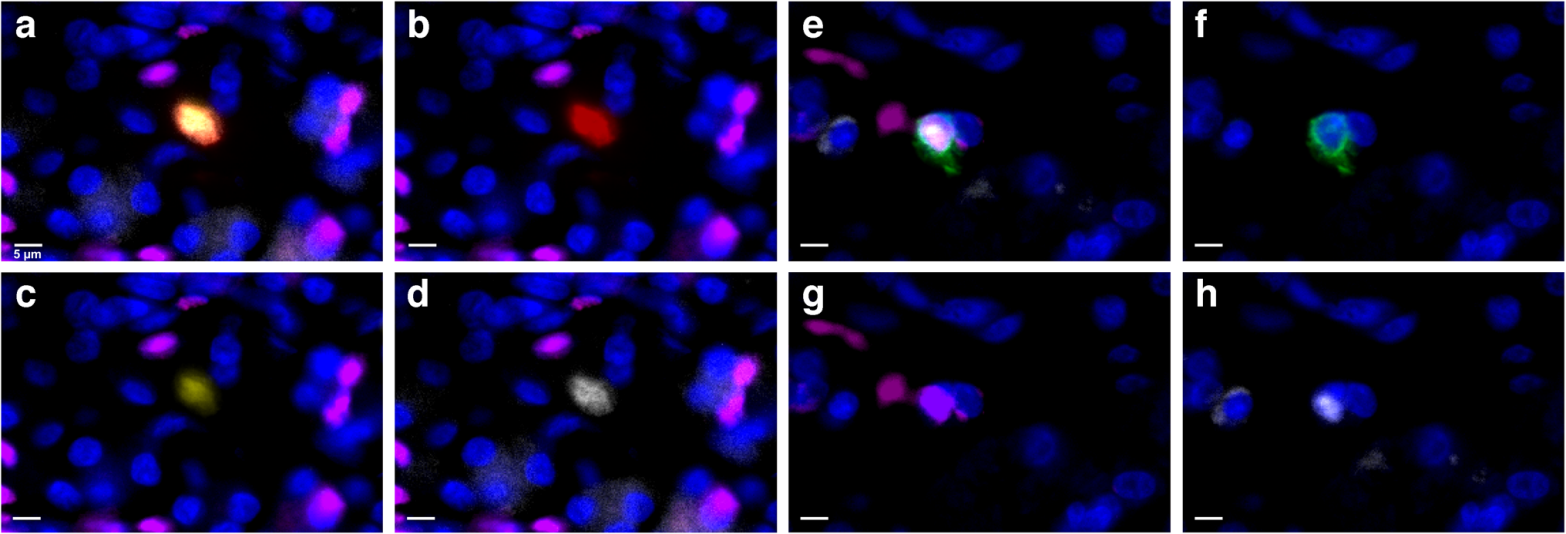
Ki67-positive extra-islet endocrine cells. Cells were stained for glucagon (red), insulin (green), ARX (yellow), PDX1 (magenta), Ki67 (white), and nuclei (DAPI, blue). (**a-d**) Representative image of a cell positive for glucagon, ARX, and Ki67. (**a**) Overlay. (**b**) Glucagon. (**c**) ARX. (**d**) Ki67. (**e-h**) Representative image of a cell positive for insulin, PDX1, and Ki67. (**e**) Overlay. (**f**) insulin. (**g**) PDX1. (**h**) Ki67. Scale bar 5 µm

Ki67-positive extra-islet endocrine cells were present in both the T1D- and control donors (0–3% of the 200 annotated cells in each donor), with no significant difference between the groups (*p* = 0.59, Table 3). One cell in the T1D group and four cells in the control group were co-positive for insulin, PDX1, and Ki67 (Fig. 3A–D). In the control group, one cell positive for only insulin and Ki67 was found as well. In the T1D group, two Ki67-positive cells that lacked hormone expression but were co-positive for PDX1 and ARX were found (Table 3). The remaining Ki67-positive cells in both groups were glucagon only, glucagon and ARX, glucagon and PDX1-positive or glucagon, and ARX- and PDX1-positive (Fig. 3E–H, Table 3). The majority of the Ki67-positive cells were intra-acinar. See Table 3 for full details of the Ki67-positive cells.

**Table 3:** The characteristics of the Ki67-positive endocrine cells. *Donor no*., donor number; *T1D*, subjects with type 1 diabetes; *CTRL*, controls; subjects without diabetes. No. of cells in group: single cells as well as small groups of up to four cells were annotated; the number of cells in this specific group (1, 2, 3, or 4 cells) is displayed. Localization: the localization of the cells was defined as: intra-acinar: located in acinar tissue, far from islets and ducts. (1) Peri-islet: being close to an islet (distance ≈ 0–3 cells) but outside of the islet perimeter. (2) Peri-ductal: being close to a duct (distance 0–3 cells) but not within the ductal epithelium. (3) Cluster: several single cells and/or groups of 2–4 cells located close together in a cluster. (4) Phenotype: markers expressed other than Ki67 (*GCG*, glucagon; *INS*, insulin). *T1D-X INS* − , area with islets devoid of insulin. *T1D-X INS* + , area with insulin containing islets

Donor no	No. of cells in group	Localization	Phenotype	
T1D-1	-	-	-	-
T1D-2	1	Intra-acinar	GCG	-
T1D-2	1	Intra-acinar	INS	PDX1
T1D-2	4	Peri-islet	GCG	-
T1D-2	4	Peri-islet	GCG	-
T1D-2	4	Peri-islet	GCG	-
T1D-2	4	Peri-islet	GCG	-
T1D-3	-	-	-	-
T1D-4 INS +	-	-	-	-
T1D-4 INS −	1	Intra-acinar/peri-ductal	PDX1	ARX
T1D-4 INS -	1	Intra-acinar	PDX1	ARX
T1D-5	1	Intra-acinar	GCG	
T1D-5	1	Intra-acinar	GCG	ARX
T1D-5	1	Intra-acinar	GCG	ARX
T1D-6	-	-	-	-
T1D-7	-	-	-	-
T1D-8	1	Intra-acinar	GCG	ARX
T1D-9 INS +	-	-	-	-
T1D-9 INS −	-	-	-	-
T1D-10	-	-	-	-
T1D-11	-	-	-	-
T1D-12 INS +	-	-	-	-
T1D12 INS −	3	Intra-acinar	GCG	PDX1
CTRL-1	1	Intra-acinar	GCG	PDX1
CTRL-1	2	Peri-islet	GCG	-
CTRL-2	2	Intra-acinar	INS	-
CTRL-2	1	Intra-acinar	GCG	ARX
CTRL-3	4	Cluster	GCG	ARX
CTRL-4	-	-	-	-
CTRL-5	1	Peri-islet	INS	PDX1
CTRL-6	1	Intra-acinar	GCG	ARX
CTRL-6	3	Intra-acinar	GCG	ARX
CTRL-7	1	Peri-islet	INS	PDX1
CTRL-7	1	Intra-acinar	INS	PDX1
CTRL-7	2	Intra-acinar	GCG	PDX1
CTRL-7	1	Intra-acinar	INS	PDX1
CTRL-8	-	-	-	-
CTRL-9	1	Peri-islet	GCG	PDX1 and ARX
CTRL-10	-	-	-	-
CTRL-11	-	-	-	-
CTRL-12	-	-	-	-

### Many extra-islet endocrine cells in both T1D and non-diabetic donors lack the expression of the transcription factors PDX1 and ARX

**Fig. 4:**
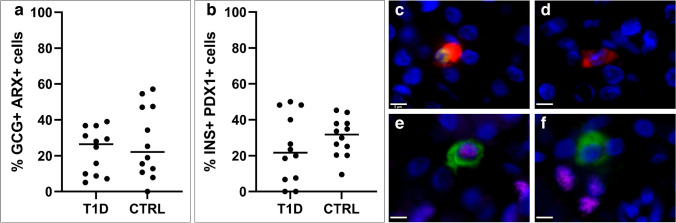
Transcription-factor expression (ARX and PDX1) in extra-islet endocrine cells. The proportion (%) of glucagon-positive cells that also express ARX (median T1D = 26.5, median CTRL = 22.1) (**a**). The proportion (%) of insulin-positive cells that also express PDX1 (median T1D = 21.7, median CTRL = 32.0) (**b**). Each dot represents an individual donor, horizontal line = median. Significance was calculated with a nonparametric Mann–Whitney test. (**c**–**f**) Cells were stained for glucagon (red), insulin (green), ARX (yellow), PDX1 (magenta), and nuclei (DAPI, blue). (**c**) A cell positive for glucagon and ARX. (**d**) A cell positive for glucagon without ARX. (**e**) A cell positive for insulin and PDX1. (**f**) A cell positive for insulin without PDX1. T1D, subjects with type 1 diabetes; CTRL, controls; subjects without diabetes. Scale bar 5 µm

Out of the glucagon-positive cells (excluding the rarer phenotypes with any combination of insulin and/or PDX1), the median proportion of glucagon-positive cells expressing ARX was 26% and 22% in the T1D and control groups, respectively (*p* = 0.6, Fig. 4a, c, and d). Out of the insulin-positive cells (excluding the rarer phenotypes with any combination glucagon and/or ARX), the median proportion of insulin-positive cells expressing PDX1 was 22% and 32% in the T1D and control groups, respectively (*p* = 0.4, Fig. 4b, e, and f). Double hormone-positive cells were found in all but four control donors, and in one T1D donor (one being donor no. T1D-4 with ICI). Other rarer phenotypes, such as INS + ARX + cells and GCG + PDX1 + cells were less frequent (Table 2). Both groups had a comparable distribution of single cells and clusters of varying cell numbers.

## Discussion

In this study, extra-islet insulin- or glucagon-positive cells in donors with, and without T1D, have been examined. In concordance with previous studies [1, 4, 5, 12, 13], we report that insulin-positive extra-islet cells are still present even after a long duration of T1D. The reduction of insulin-expressing extra-islet cells was seemingly less pronounced than the loss of beta cells within islets, as extra-islet insulin-positive cells were found in all but one T1D donor. This implies that the extra-islet insulin-positive cells has a capacity to be replenished, and/or that they have a higher resistance to the events causing the beta-cell loss in T1D. Although the extra-islet insulin-positive cells were often present, they were reduced compared to donors without diabetes. This could be a consequence of the same beta-cell destructive process(es) causing the primary demise of islet beta cells in T1D or secondary to hyperglycemia-associated glucose toxicity [4, 5]. In this context, the three donors with remaining ICI in some lobes are of particular interest. A more overarching pathological mechanism, such as glucose toxicity, would likely cause a widespread and equal demise of beta cells in the pancreas. However, in all three donors, there were 2.5 to 15 times more extra-islet insulin-positive cells in the areas where ICI was present, compared with in the areas with IDI. This argues against a system-wide pathological mechanism of beta cell demise in T1D, and for a selective one that affects most, but not all, lobes.

The increased frequency of extra-islet glucagon-positive cells in T1D is noteworthy, and the frequency is considerably higher compared to what has been previously reported for islets (median 53.0 extra-islet cells/mm^2^ in T1D, median 11.2 extra-islet cells/mm^2^ in controls, and median 4.4 islets/mm^2^ in control subjects) [14]. Importantly, the increased frequency was maintained even when glucagon-positive cells in the peri-islet and peri-ductal areas were excluded (data not shown), confirming that the increased glucagon frequency was not merely an effect of, e.g., shattered islets. There are several possible explanations for the increased number of glucagon-positive cells. The reduced pancreas size observed in T1D [15, 16], possibly resulting from loss of acinar but not endocrine cells in the exocrine parenchyma, may contribute to the observed increased frequency of glucagon-positive cells but is unlikely to explain the entire increase that was almost fivefold. Islet alpha cells have been shown to have a reduced function in T1D, with impaired glucagon secretion and altered gene expression [17]. The increase of glucagon-positive cells scattered in the exocrine parenchyma could be a compensatory response to counterbalance the impaired alpha-cell function.

Conversion of alpha cells into beta-cells upon GABA exposure, as a form of beta-cell neogenesis, has been debated but elegantly shown in a mouse model as well as in human islets transplanted into mice [18, 19]. This, in turn, triggered an alpha cell replacement mechanism through neogenesis from ductal precursor cells [18]. The increased number of glucagon-positive extra-islet cells observed herein could suggest that this mechanism of alpha-cell replacement is functional in donors with T1D. Conversion of human alpha-cells into beta-cells has been observed in vitro as well [20]. Furthermore, in this study, we observed a lack of transcription-factor expression in many cells, suggesting that they could be immature, newly formed or plastic, in line with the idea of neogenesis and/or conversion of cells. An alternative hypothesis would be that the increased number of glucagon- and decreased number of insulin-positive extra-islet cells are the result of trans-differentiation of beta cells to alpha cells as shown in experimental studies and in T2D [21–23]. However, the mechanism of conversion has also been questioned [19]. In line with this, intermediate phenotypes such as cells co-positive for glucagon and PDX1, which have been described in the context of in vitro transdifferentiation [20], were very rarely found in this study.

Several mechanisms for the expansion of beta-cell mass in non-diabetic settings have been proposed [24–27]. In the current study, we report a mitotic activity of between 0 and 3% in both donor groups, defined as Ki67-positive expression in the nuclei. However, only 200 cells were examined per donor, and out of these a very limited number of cells showed mitotic activity, adding a level of uncertainty to the results. Nevertheless, the observed mitotic activity is high in comparison to what have been reported for islets [28–30], suggesting the extra-islet cells could play a role in the endocrine cell expansion.

In the current study, we characterized the extra-islet endocrine cells in T1D by examining well-preserved pancreatic tissue obtained from heart-beating organ donors previously diagnosed with T1D. However, the sections were only analyzed in 2-D which has the possible consequence that some annotated single cells could be part of the outermost border of an islet. Yet, in a previous study on extra-islet single cells, this possibility was addressed by examining consecutive sections, and it was concluded that no single cells were part of an islet [31]. An additional constraint of the study is that only a limited area of each pancreas was investigated, as well as relatively few cells. However, samples from only the body/tail of the pancreas were deliberately chosen to avoid confounding factors from the islets originating from the uncinate process and the head of the pancreas. Despite the above mentioned limitations, this study represents, to the best of our knowledge, the largest investigation to date conducted on extra-islet cells in T1D.

In summary, we provide a characterization of extra-islet cells expressing insulin or glucagon in donors with or without T1D. Results presented suggest that the pathological mechanisms involved in T1D not only affect islets regionally, but also the extra-islet beta-cells in affected lobes of the pancreas. Surprisingly and of high interest, we also present an increase in the frequency of extra-islet glucagon-positive cells in donors with longstanding T1D. The presence of extra-islet insulin- and glucagon-positive cells with mitotic activity suggests preserved renewal of endocrine cells in donors both with and without T1D.

## Supplementary Information

Below is the link to the electronic supplementary material.

Supplementary file1 (DOCX 402 KB)

## Data Availability

The datasets generated during and/or analyzed during the current study are not publicly available, but are available from the corresponding author on resonable request.
