# Supplementary material for: Loss of insulin-expressing extra-islet cells in type 1 diabetes is accompanied with increased number of glucagon-expressing extra-islet cells
Source: Virchows Arch. 2024 Jun 26;486(4):687–95. doi: 10.1007/s00428-024-03842-4 (PMC12018523; doi:10.1007/s00428-024-03842-4)
Supplement: Supplementary file 1 — Supplementary file1 (DOCX 402 KB) [file 428_2024_3842_MOESM1_ESM.docx]

Supplementary Figures and Tables

| **Antibody/OPAL** | **Species** | **Concentration** | **Diluted in** | **Catalog #** | **Company** | **Matched with:** |
| --- | --- | --- | --- | --- | --- | --- |
| **PDX1** | Goat | 5 µg/ml | Blocking buffer | AF2419 | R&D systems | OPAL 520 |
| **Insulin** | Guinea Pig | Ready-to-use |  | IR00261-2 | Agilent | OPAL 570 |
| **Glucagon** | Rabbit | 1:500 | Blocking buffer | AC-0079 | Epitomics | OPAL 620 |
| **ARX** | Sheep | 5 µg/ml | Blocking buffer | AF7068 | R&D systems | OPAL 690 |
| **Ki67** | Mouse | 1:50 | Blocking buffer | M724029-2 | Agilent | OPAL 480 |
| **ImPress Rabbit (and Guinea Pig)** |  | Ready-to-use | Blocking buffer | K4003 | Dako |  |
| **ImPress Mouse** |  | Ready-to-use | Blocking buffer | K4001 | Dako |  |
| **Goat Sec** | Donkey | 1:2000 | Blocking buffer | Ab6885 | Abcam |  |
| **Sheep Sec** | Donkey | 1:2000 | Blocking buffer | Ab6900 | Abcam |  |
| **OPAL 480** |  | 1:200 | Amplification diluent | NEL821001KT | Akoya Biosciences |  |
| **OPAL 520** |  | 1:200 | Amplification diluent | NEL821001KT | Akoya Biosciences |  |
| **OPAL 570** |  | 1:200 | Amplification diluent | NEL821001KT | Akoya Biosciences |  |
| **OPAL 620** |  | 1:150 | Amplification diluent | NEL821001KT | Akoya Biosciences |  |
| **OPAL 690** |  | 1:150 | Amplification diluent | NEL821001KT | Akoya Biosciences |  |

**Supplementary table 1.** **Primary antibodies, secondary antibodies, and Opals used for multiplex staining.** The staining was performed in the order in which the antibodies appear in the table


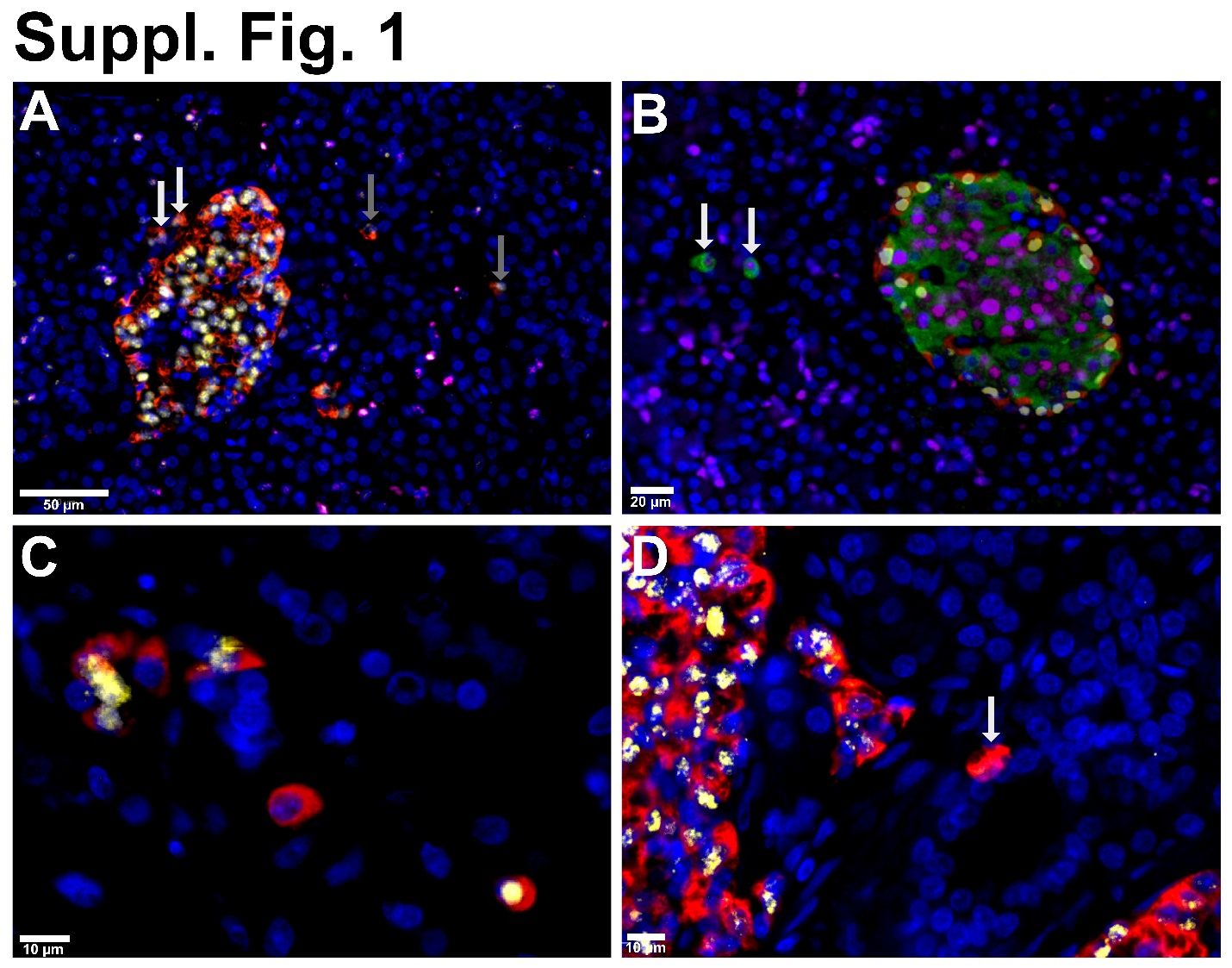


**Supplementary Figure 1. Representative images of the 4 different locations. a)** Peri-islet (white arrows) and Intra-acinar: extra-islet cells found close to an islet, but outside of the islet border, as well as in the acinar tissue (grey arrows). **b)** Intra-acinar: extra-islet cells surrounded by acinar tissue and not being defined as peri-islet or peri-ductal (white arrows). **c)** Several single cells and/or groups of 2-4 cells located close together in a cluster (but not in direct contact with each other). **d)** Peri-ductal: being close to a duct but not within the ductal epithelium (white arrow)

**Autostainer protocol.** The staining protocol, including deparaffinisation, was run on the Autostainer BOND RX System from Leica Biosystems (21.2821). The protocol for the autostainer is found below.

1. Bond Dewax Solution (Leica, AR9222) x 3 at 72°C

2. Reagent Grade Alcohol (≥90 %) x 3

3. Bond Wash Solution (Leica, AR9590) x 5

4. Epitope Retrieval Solution 1 (ER1, pH 6, Leica, AR9961) for 20 min at 95°C (not

used for antibody 1)

5. Bond Wash Solution x 3

6. PKI Blocking Buffer (Akoya Biosciences, NEL821001KT) for 5 min at RT

7. Primary Antibody, incubation time 30 min (Ab 2, 3 and 5) or 40 min (Ab 1 and 4)

8. Bond Wash Solution x 3

9. Secondary Antibody, HRP conjugated, incubation time 10 min

10. Bond Wash Solution x 5

11. Opal Fluorophore, incubation time 10 min

12. Bond Wash Solution x 4

Repeat steps 4-12, staining the next antibody, until all 5 antibodies are done.

13. Real Dapi (Akoya Biosciences, NEL821001KT) for 5 min

14. Bond Wash Solution x 4
